# Hepatocellular Carcinoma: Molecular Mechanisms and Targeted Therapies

**DOI:** 10.3390/medicina55090526

**Published:** 2019-08-23

**Authors:** Ali Alqahtani, Zubair Khan, Abdurahman Alloghbi, Tamer S. Said Ahmed, Mushtaq Ashraf, Danae M. Hammouda

**Affiliations:** 1 Department of Internal Medicine, College of Medicine and Life Sciences, University of Toledo, Toledo, OH 43614, USA; 2 Division of Gastroenterology and Hepatology, Health Science Center at Houston, The University of Texas, Houston, TX 77030, USA; 3 Division of Hematology and Medical Oncology, College of Medicine and Life Sciences, University of Toledo, Toledo, OH 43614, USA

**Keywords:** hepatocellular carcinoma, molecular pathways, targeted therapy, precision medicine

## Abstract

Hepatocellular carcinoma (HCC) is one of the most common and lethal malignant tumors worldwide. HCC is a complex process that is associated with several etiological factors, which in turn result in aberrant activation of different cellular and molecular pathways and the disruption of balance between activation and inactivation of protooncogenes and tumor suppressor genes, respectively. Since HCC most often occurs in the setting of a diseased or cirrhotic liver and most of the patients are diagnosed at the late stage of disease, prognosis is generally poor. At present, limited treatment options with marginal clinical benefits are available. Systemic therapy, particularly in the form of conventional cytotoxic drugs, are generally ineffective. In recent years, molecular-targeted therapies have been clinically used to treat various cancers, including liver cancer. This approach inhibits the growth of tumor cells by interfering with molecules that are involved in carcinogenesis, which makes it more selective and specific than cytotoxic chemotherapy. Many clinical trials have been carried out while using molecular targeted drugs in advanced HCC with many more in progress. The clinical trials in HCC to date have evaluated a single-targeted therapy alone, or two or more targeted therapies in parallel. The aim of this review is to provide insight of various molecular mechanisms, leading to HCC development and progression, and also the range of experimental therapeutics for patients with advanced HCC. The review will summarize different clinical trials data the successes and failures of these treatments, as well as the most effective and approved drugs designed against HCC.

## 1. Introduction

Liver cancer is the fifth most common type of cancer and it is the second most common cause of cancer related mortality globally [1,2,3,4], with an estimated 746,000 deaths in 2012 [5]. The incidence of liver cancer and mortality shows a stable increase worldwide. An estimated incidence of primary liver cancer ranges from 600,000 to 800,000 annually, accounting for 5.6% of all human cancers and projected cases of about a million by 2030 [1,2,6]. Liver cancer consists of a heterogeneous group of malignant tumors with varied histological characteristics and unfavorable prognosis [2]. The major hepatocellular neoplasms include hepatocellular carcinoma (HCC), intrahepatic cholangiocarcinoma (iCCA), hepatoblastoma, hepatocellular adenoma, and the pediatric neoplasm [1,2]. HCC is the most common and iCCA is the second most common primary liver cancers [2,7].

### Hepatocellular Carcinoma: Incidence, Risk Factors, Prognosis

Hepatocellular carcinoma that originates in the liver accounts for about 80%–90% of all primary liver cancers [1,2]. HCC incidence and mortality rates have been rising for decades with almost 800,000 new cases occurring every year [2]. HCC has a wide geographic variability with predominance in developing countries; more than 80% of HCC occur in Asia and sub-Saharan Africa [8]. Nonetheless, the incidence of HCC is rising in the United States (US) and other developed countries [1], and during the past 20 years HCC has been increased by 114% in the US [8]. The global distribution patterns of HCC reflect geographical variation, ethnic disparities, specific etiological factors, and socioeconomic status [9,10]. 

HCC is highly fatal disease, which usually occurs as a consequence of underlying liver dysfunction. In the majority of cases, cirrhosis of liver precedes the development of HCC [3,11]. Various risk factors (both environmental and genetic) have been associated with HCC, including chronic infection with Hepatitis B (HBV) and Hepatitis C (HCV) viruses, excessive alcohol intake, consumption of food stuffs infected with fungal toxin-aflatoxin B1 (AFB1), nonalcoholic fatty liver disease, diabetes, obesity, tobacco use, and hereditary hemochromatosis [3,11]. Among them, HBV or HCV infection are the principal causative agents for HCC worldwide [12]. The risk factors vary in different geographical regions, leading to variation in global distribution patterns [11,12]. In Asia (particularly China) and Africa, between 40% and 90% of HCCs result from chronic HBV infection [12]. Chronic HCV infection develops into liver cirrhosis in 20% cases and among them 2.5% normally develop HCC [10]. In Singapore, Japan, and Australia, high incidence of HCV infection results in an increased occurrence of HCC. Moreover, in Europe and in the US, HCV infection is considered to be the leading cause of HCC [12]. The anticipated rate of HCC development from chronic hepatitis B and C is 0.5%–5% per year [8]. AFB1 is the predominant etiological factor for HCC in certain regions of Africa and Asia [12].

HCC is usually diagnosed at an advanced and unresectable stage, when palliative therapies are employed with a median survival of 6–12 months following diagnosis [5,8]. In the US, the two-year survival is less than 50% and five-year survival is only 10% [5]. Curative treatment options for early-stage HCC include: surgical resection, radiofrequency/microwave ablation, transarterial chemoembolization, liver transplantation, and rarely systemic chemotherapy [5]. Nevertheless, the main drawbacks of curative treatment are recurrence of HCC, which leads to an incidence of more than 70% at five-year [4], and the unavailability of properly matched donors for liver transplantation [5]. Moreover, systemic chemotherapies in unresectable and recurrent cases with underlying liver dysfunction are ineffective, with low survival benefits [4,7], as the patients fail to withstand the trials of new chemotherapeutic agents, in part due to underlying liver dysfunction [13]. Hence, there is increased need for effective alternative treatment strategies in patients with advanced or metastatic HCC. Molecular targeted therapy based on the molecular pathways that lead to carcinogenic mechanisms of HCC is a novel and promising treatment approach. A proper understanding of the molecular mechanisms of hepatocarcinognesis and identification of appropriate target molecules and signaling pathways responsible for tumor phenotype is crucial in order to develop effective targeted therapies against HCC [8,13].

The purpose of this review is twofold: firstly, to discuss the molecular pathogenesis and signal transduction pathways that are involved in HCC development and secondly to discuss the novel molecular-targeted therapeutic agents showing promising results in clinical trials for the treatment of HCC.

## 2. Cancer Biology

### 2.1. Fundamentals of Carcinogenesis

Cancer development is a multi-step process that transforms normal cells into invasive cancer cells via pre-neoplastic states. The basic features of cancer cells include: uncontrolled cell proliferation, immortality, genomic instability, and capacity to disrupt local and distant tissues (metastasis) [14]. Cancer cells are self-sufficient, as they produce their own growth signals (autocrine stimulation), remain insensitive to growth-inhibitory signals, are resistant to apoptotic signals, and can perform angiogenesis [13,14]. Genomic instability in cancer cells results from an accumulation of mutations in DNA, which can be via germline mutation and/or spontaneous somatic cell mutations. The majority of malignancies result from somatic mutations, which are triggered by various endogenous and environmental factors, including exposure to mutagens, viral infection, and diet [8]. There are three main phases in the process of carcinogenesis: initiation, promotion, and progression [15]. Tumor initiation occurs due to early mutations, and a wide array of further changes are responsible for tumor progression [8]. The tumor suppressor genes and proto-oncogenes are two main categories of genes that are typically altered in cancer. A disruption in balance between the activation and inactivation of these two types of genes is considered to play key role in cancer development [16]. 

### 2.2. Sequential Development of HCC

**Figure 1:**
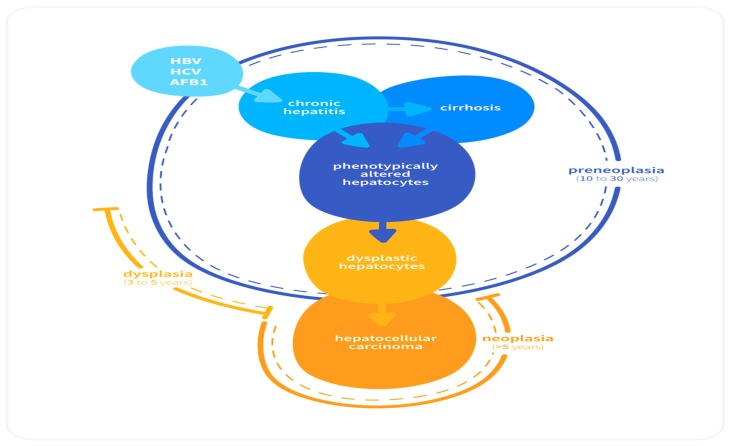
Sequence of cellular lesions in liver leading to the development of hepatocellular carcinoma [18].

Similar to other neoplasia, the development of HCC is a complex, multistep process that stems from a combination of genetic and environmental factors [8]. Irrespective of etiology, cirrhosis precedes HCC in a majority of the patients. The regenerating nodules that are produced during cirrhosis provide a favorable microenvironment for the transformation of normal hepatocytes to dysplastic hepatocytes to neoplastic lesions and culminating in HCC (Figure 1) through the subsequent accumulation of genetic and epigenetic changes [17,18]. 

### 2.3. Role of Inflammation in HCC

To date, the sequential development of hepatocarcinogenesis starting from preneoplastic lesion to dysplastic hepatocytes and finally hepatic neoplasm is not completely understood. It is assumed that the entire process of hepatocarcinogenesis involves the collaborative action of several cellular mechanisms such as change in tumor microenvironment, necroinflammation, oxidative stress, and hypoxia, along with other molecular mechanisms, including the transcription and activation of cytokines, chemokines, and growth factors, DNA damage, and DNA methylation [19]. A multitude of clinical and epidemiological studies revealed a strong correlation between inflammation and cancer development. HCC is one of the more extensively researched inflammation-related carcinogenesis, as more than 90% of HCCs arise in the context of hepatic injury and inflammation [19]. Chronic liver infection that is caused by HBV or HCV, or exposure to aflatoxins or alcohol, causes persistent hepatic injury and hepatocyte cell death and simultaneous hepatocyte regeneration, which thereby triggers deregulated hepatocyte proliferation and subsequent hepatic inflammation [20]. In the pre-malignant environment, the inflammatory cells release a multitude of cytokines, chemokines, growth factors, prostaglandins, and proangiogenic factors, making the hepatic milieu a favorable zone for hepatocyte transformation by an accumulation of genetic mutations. The survival of transformed hepatocytes is possible by the activation of anti-apoptotic pathways and the suppression of immune surveillance [21]. A complex interplay of different pro-inflammatory (such as Interleukin-6, or IL-6, Tumor Necrosis Factor, or TNF-α) and anti-inflammatory cytokines (Transforming Growth Factors α and β or TGF-α and β), different transcription factors (NF-κβ, STAT-3), and their signaling pathways are involved in HCC development [19,20]. 

#### 2.3.1. IL-6 and TNF-α

**Figure 2:**
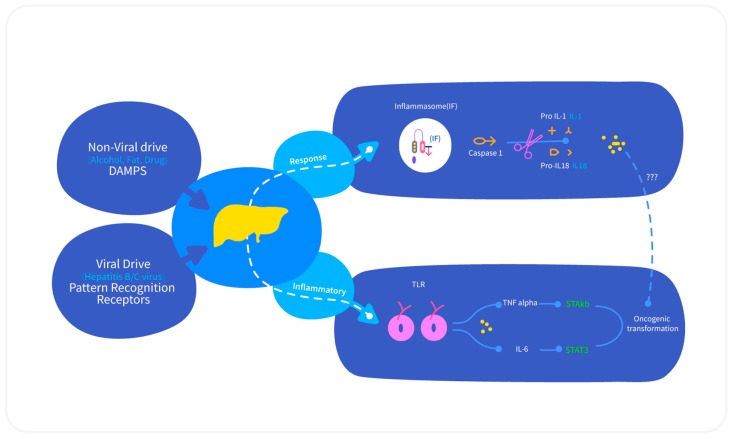
Basic molecular events during inflammatory hepatocarcinogensis. The inflammatory response caused by viral (microbial attack) or non-viral etiologies (sterile attacks) produced proinflammatory cytokines through inflammasome-dependent or independent pathways. The inflammosome component provides a platform for activation of caspase. Proinflammatory cytokines, through activation of transcription factors or by some unknown mechanisms make the hepatic environment suitable for cellular transformation. The accompanying pathological stages are shown in right panel. DAMPS—damage-associated molecular patterns [17].

The expression of IL-6 and TNF-α during chronic hepatic injury activates downstream targets of STAT3 transcription factor, which drives neoplastic transformation in the liver microenvironment [20] (Figure 2). Further, TNF-α promotes hepatic tumor growth and HCC recurrence. A recent study by Jing et al. [22] revealed that the overexpression of TNF-α promotes HCC through the activation of hepatic progenitor cells (HPCs) and the knocking down of TNF-α inhibited HPC activation and proliferation, which reduces tumor incidence. This confirmed that TNF-α plays significant role in liver injury and prognosis.

#### 2.3.2. Nuclear Factor-κβ (NF-κβ) 

Nuclear factor-κβ (NF-κβ) is a master transcriptional regulator of inflammatory response and cell death [23]. A number of studies substantiated the role of NF-κβ in the development of hepatocellular injury, liver fibrosis, and HCC. Activated NF-κβ is a frequent and early event in HCC, irrespective of etiology and it is linked with the attainment of a transformed phenotype during hepatocarcinogenesis. Therefore, NF-κβ is proposed to be a central link between hepatic injury, fibrosis, and HCC [17,23].

#### 2.3.3. TGF-α

TGF-α—a polypeptide that promotes cellular proliferation and transformation, has thought to bear a close relationship with hepatocarcinogenesis. In normal liver cells, there is a low expression of TGF-α. Combined actions of different cytokines secreted as a chronic inflammatory response following hepatic injury persistently upregulate TGF-α in the liver [18], and consequently allow the regeneration of hepatocytes, hepatocyte proliferation, dysplasia of hepatocytes, and finally the development of HCC [24]. TGF-β is also up-regulated in HCC and it plays a critical role in HCC progression by inducing tumor cell migration and invasion [25].

## 3. Molecular Events in HCC

With the advent of different molecular biology techniques, we are able to unravel the molecular mechanisms of tumorigenesis more quickly and propose new theories of carcinogenesis, which may ultimately lead to an improvement in treatment options. Despite these recent advancements, the molecular pathogenesis of HCC is not yet fully elucidated at this time [16]. What is known is that the development of HCC involves the accumulation of multiple genetic and epigenetic changes during the initiation, promotion, and progression steps [11]. The most frequent types of molecular aberrations in HCC are:

(i) Telomere shortening
(ii) Copy number variants
(iii) Single nucleotide variants and small deletions
(iv) Epigenetic modifications

### 3.1. Telomere Shortening

Alterations of gene expression can occur due to point mutations and chromosomal aberrations. Chromosomal loss or gain (deletions or amplifications) are detected in most cases of HCC; the most prevalent being amplifications of 1q (58%–78%), 6p, 8q, 17q, and 20q and deletions in 1p, 4q, 5q, 6q, 8p, 13q, 16q, and 17p [8,26]. Chromosomal losses occur in 25%–40% of HCCs, whereas chromosomal gains occur in 30%–55% cases [8]. Increased amplification has been observed in 11q13 regions encoding cyclin D1 and 6p21 regions encoding VEGFA [26]. 

### 3.2. Copy Number Variants

In a majority of cancers, mutations occur in two key classes of genes—the tumor suppressor genes and proto-oncogenes [8]. In normal cells, the tumor suppressor genes are expressed in a low level and they are responsible for preventing tumor growth by inhibiting cellular proliferation and inducing terminal differentiation and cell apoptosis. They are recessive genes, and thus require a loss of function of both alleles to generate a mutant phenotype [8,16]. The most common tumor-suppressor genes mutated in cancer are *p53*, *pRb*, *p21*, and *PTEN* [16]. 

In contrast, proto-oncogenes control cellular proliferation and they are expressed in a very low level in normal cells. They encode proteins that are an integral part of cellular signal transduction pathways. Genetic mutations of proto-oncogenes transform them into constitutively active oncogenes, which then may initiate carcinogenesis. Unlike tumor-suppressor genes, the proto-oncogenes are dominant genes and the mutation of one allele is sufficient to cause a mutant phenotype. Besides mutation, the genes can also be amplified and then overexpressed to lead to carcinogenesis [8]. *Ras*, *c-fos*, *c-erb2*, and *c-myc* are the most common proto-oncogenes that are mutated in human cancers. Overall, proto-oncogenes seem to be less important in HCC pathogenesis when compared to tumor suppressor genes (8p).

#### 3.2.1. p53

**Figure 3:**
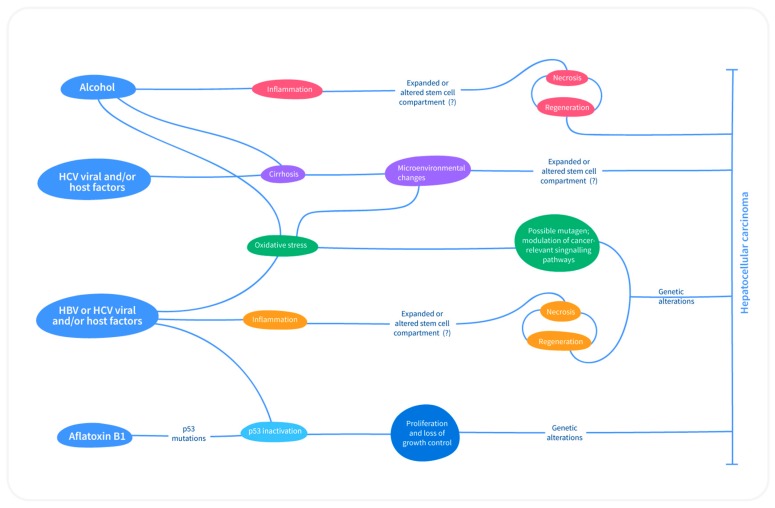
Mechanisms of hepatocarcinogenesis. The suspected mechanisms of hepatocarcinogenesis for various risk factors. Same color indicates commonalities. Hepatitis B virus (HBV) and aflatoxin both can affect the genome—HBV can integrate into host genome and aflatoxin B1 is a mutagen. Hepatitis C virus (HCV) cannot integrate into the host genome [10].

p53 is a tumor suppressor gene that is located on chromosome 17p and it encodes a 53 KD DNA-binding transcription factor [8]. In normal cells, p53 is responsible for regulating cell cycle progression, DNA repair, and apoptosis [8,11]. The expression of p53 increases in response to cellular stress and DNA damage. Usually, a single point mutation of one allele and the deletion of other allele inactivates p53 [8]. The loss of p53 is a major driver of HCC progression, irrespective of etiology. Mutations of p53 has been observed in 30%–60% HCCs, and a plethora of clinical studies reported that the most common p53 mutation is G to T transversion in codon 249, and very rarely G to C tranversion [11]. The oxidative stress also causes p53 mutations; mostly from G:C to T:A transversion at codon 249 and from C:G to T:A or from C:T to A:T at codon 250, which increases the risk of HCC development 200-fold [11]. The inactivation of p53 is associated with some of the etiological factors of HCC. Exposure to AFB1 consistently results in p53 G to T transversion, thus inactivating p53; in fact, the risk of HCC development is proportional to the amount of ingested AFB1 [8,26]. Since AFB1 contamination is predominant in Africa and Asia, codon-specific G to T transversion of p53 gene has been found in 50%–100% HCC cases in Asia and Africa and rarely found in US cases [8]. AFB1 exposure is directly associated with tumor initiation without the development of cirrhosis, which suggests that AFB1 primarily drives HCC development. Additionally, HCV viral protein NS5A is known to interact and suppress p53 by sequestration to the perinuclear membrane, thereby affecting p53 mediated cell cycle regulation, apoptosis, and responses towards cellular stress [10] (Figure 3). 

#### 3.2.2. pRb

pRb—Retinoblastoma protein pRb1 controls cell cycle progression and prevents tumor development and its inactivation deregulates cell cycle progression, which causes uncontrolled cell proliferation. pRb controls the activity of various cyclin-dependent kinases (CDKs) that are involved in G1/S cell cycle transition. Altered expression of a number of CDK inhibitors, such as p16^INK4A^, p21^WAF1/C1P1^, and p27^Kip1^ (either one or more), occur in almost 90% HCC cases. p16^INK4A^ remains inactivated during both early and late stages of hepatocarcinogenesis. Many studies showed severe disruption of pRb pathway in HCC, including altered pRb expression and the loss of pRb [11]. 

#### 3.2.3. Ras

Ras family (*H-ras*, *K-ras*, N-ras) are proto-oncogenes that transduce myogenic signals to mitogen-activated protein kinases (MAPK1) MEK1 and MEK2 through serine/threonine kinases Raf1 [27] and stimulate cell growth, differentiation, and apoptosis [11]. The *ras*-family proto-oncogenes are activated by single point mutations at codon 12 for *N-ras*, 13 of *H-ras*, 61 and 64 of *K-ras* [11]. The mutation rate of the *ras* family gene is quite low and rare in human HCC [11,27], although one study reported that 30% of HCCs might have *ras* mutations [28].

#### 3.2.4. c-myc 

Proto-oncogene *c-myc* is involved in cell growth and differentiation. In normal liver cells, *c-myc* expression is low to almost none, whereas in most human hepatoma cell lines, *c-myc* is overexpressed. In vivo studies revealed a progressive rise of *c-myc* level from normal liver to chronic hepatitis, cirrhosis, and HCC. *c-myc* is overexpressed predominantly through gene amplification in 40%–60% of HCC or by promoter hypomethylation [8]. The overexpression of *c-myc* was observed in the early stages of human HCC and some studies depict a strong correlation between *c-myc* activation and malignant conversion of preneoplastic, high grade dysplastic liver nodules into cancerous cells. The findings of different studies indicate that the overexpression of *c-myc* during the early stages of HCC plays a central role in malignant transformation [27]. 

#### 3.2.5. c-fos Activation

The proto-oncogene *c-fos* is an important member of activating protein-1 (AP-1) transcription factor responsible for cellular transformation, proliferation, differentiation, and apoptosis. *c-fos* is required in all phases of cell cycle. The overexpression of *c-fos* had been detected in HCC and one study revealed that hepatocytes overexpressing *c-fos* proliferate continuously, even in the absence of growth factors [29].

#### 3.2.6. ErbB Receptor Family

The ErbB family of receptor tyrosine kinases comprises of four members (ERBB1-ERBB4). The overexpression of ERBB1 (also known as EGFR) is detected in 68% HCC cases, ERBB3 in 61%, ERBB2 (also called Her2) in 21%, and ERBB4 in 61% HCC cases. Moreover, the overexpression of ERBB1 and ERBB3 is linked with more aggressive tumor with high proliferation index, intrahepatic metastasis, de-differentiation, and tumor size [10].

#### 3.2.7. Single Nucleotide Variants and Small Deletions

Genomic instability may result from telomerase shortening, abnormal methylation, and/or aberrations in mismatch repair genes. Telomere shortening is an essential characteristic of chronic hyper-proliferative liver disease, which, in combination with hepatocyte turnover, induces genomic instability, which leads to HCC [10]. A hypothesis is that telomere shortening pushes chromosomal instability and cancer-promoting lesions during early phases of hepatocarcinogenesis and telomerase re-activation induces malignant progression [10]. In 90% HCC, the overactivation of telomerase enzyme is detected. Telomerase activity is associated with HBV infection, since HBV integrates in the telomere reverse transcriptase (TERT) locus. Moreover, the amplification of telomerase RNA component (TERC) gene and allelic loss of chromosome 10p region encoding telomerase repressor affects telomerase activity [26].

#### 3.2.8. Epigenetic Alterations

The aberrant methylation of regulatory regions (particularly promoters) of genes causes the epigenetic silencing of gene expression [26]. An abnormal DNA hypermethylation pattern over a background of global hypomethylation has been identified in human HCC. Typically, methylation occurs in the initiation and progression stages of hepatocarcinogenesis [10]. Promoter hypermethylation and the silencing of some tumor suppressor genes, such as *p16^INK4A^*, *E-cadherin*, *BRCA1*, *IGFR-II*/*MP6*, and *COX-2*, occur in HCC [10]. 

### 3.3. Etiologic Factors and Associated Molecular Mechanisms in HCC

#### 3.3.1. Viral Induced HCC

Hepatocarinogenesis that is driven by HBV and HCV infection has complicated mechanism involving both host and viral factors.

##### HBV Infection

HBV is a partially double-stranded non-cytopathic DNA virus that belongs to the *Hepadnaviridae* family [10]. Following HBV infection, there is hepatocyte injury, chronic necro-inflammation, hepatocyte proliferation, fibrosis, and eventually cirrhosis. A higher rate of hepatocyte turnover in cirrhosis along with accumulation of mutations in the host genome may lead to genetic alterations, chromosomal aberration, activations of oncogenes, and inactivation of tumor suppressor genes. Additionally, HBV infection can directly cause HCC without antecedent cirrhosis. The integration of HBV into host genome results in chromosomal rearrangement, thereby enhancing genomic instability [30] (Figure 3). Moreover, HBV encodes a regulatory protein (HBx), which transactivates certain genes that are involved in the regulation of cell proliferation, such as Ras, Raf, MAPK, ERK, and JNK [10,30]. Furthermore, HBx binds and suppresses genes that are involved in cell cycle control, cellular DNA repair, and apoptosis, such as p53. Ninety percent of HBx transgenic mice found to develop HCC, thus corroborating the hepatocarcinogenic potential of HBx [10]. Moreover, AFB1 synergistically works with HBV infection and studies determined a 5 to 10-fold higher risk of development of HCC with simultaneous exposure to AFB1 and HBV, rather than exposure to only one of these factors [8,10]. This cooperative effect may arise due to mutagenesis that is induced by AFB1 and persistent hepatocyte death and regeneration following chronic HBV infection [10].

##### HCV Infection 

HCV is an RNA virus that belongs to *Flaviviridae* family. Unlike HBV, HCV is unable to integrate into the host genome. Thus, it causes HCC indirectly with antecedent cirrhosis as a hallmark (Figure 3). In general, the pathogenic interactions between immune system and HCV-induced HCC are extremely complicated and they require further elucidation. One theory for HCV-induced hepatocarcinogenesis is that immune response towards virus results in continuous cycle of hepatocyte death and regeneration, causing the constant accumulation of genetic mutations, leading to tumor formation [10]. Moreover, the core HCV proteins, such as NS5A and NS3 induce oxidative stress, which activates NF-κβ and MAPK signal transduction pathways, thereby upregulating some of the genes responsible for pro-inflammatory cytokine production, consequent inflammation, alterations in apoptotic pathways, cell proliferations, and tumor formation [30]. Additionally, excessive alcohol intake has been found to be correlated with higher HCV infection, and the combined effect of alcoholism and HCV infection lead to higher rates of cirrhosis and HCC as compared to nondrinkers [30]. The additive effects and the exact mechanism by which alcohol aggravates HCV-related disease are not quite clear; however, impaired immune response, increased viral replication, and higher hepatocyte toxicity are considered to be the main factors that lead to progressive hepatic disease [31]. In HCV-infected patients, increased hepatocyte apoptosis or programmed cell death has been observed [32], which is anticipated to occur as histoimmune response mediated via cytotoxic T lymphocytes and natural killer cells for viral clearance. Caspases are the enzymes that cause cell death [31]. A protooncogene *Bcl-2* acts as an apoptotic inhibitor by blocking the action of caspases. Studies revealed that the synergistic effect of HCV infection and alcohol consumption leads to the alteration of viral genome, downregulation of *Bcl-2* expression, resulting in higher rate of apoptosis, and aggressive cirrhosis [30,31,32]. Moreover, the combined effect of alcohol and HCV infection promotes severe oxidative stress, producing reactive oxygen species, releasing pro-inflammatory cytokines, particularly TNF-α, resulting in chronic hepatocyte destruction and regeneration, along with stellate cell activation, cirrhosis, and ultimately HCC [10,30] (Figure 3).

#### 3.3.2. Nonalcoholic Fatty Liver Disease and HCC 

Lately, Nonalcoholic fatty liver disease (NAFLD) becomes one of the leading causes of HCC. A systematic review by White et al. [33] reported that the annual incidence rate for developing HCC in patients with NASH-related cirrhosis is approximately 2.4%–12.8%. Many risk factors, such as genomic instability, insulin resistance, and immune activation, are hypothesized to play a role in altering the signaling pathway of NASH patients and lead to HHC [34]. While the mechanism of NASH-related HCC is not fully understood, but the emerging evidence, suggesting the role of hyperinsulinemia secondary to insulin resistance, leads to increased expression of the insulin-like growth factor-1 (IGF-1), which triggers signaling cascade via insulin receptor substrate-1 (IRS-1) and eventually activate the PI3K and MAPK pathways [35]. The activation of the PI3K and MAPK pathways has a noticeable role in developing dysplastic hepatocytes by increased cell proliferation and inhibition of apoptosis [36].

#### 3.3.3. Hemochromatosis and HCC 

Hereditary hemochromatosis (HH), a metabolic oxy-radical disorder, is known to be linked with cirrhosis and HCC. The majority of HH patients will first develop cirrhosis and about 40%–60% of them finally develop HCC [8]. In fact, the risk for patient development of HCC in with HH is 200-fold higher than patients with other types of cirrhosis [8,33]. Iron overload in the liver in the setting of HH might cause liver damage and subsequent hepatocarcinogenesis by generating abundant reactive oxygen/nitrogen species, which can damage DNA and mutate cancer-related genes [12,37].

### 3.4. Different Cellular Signaling Pathways Linked to HCC

Growing research on tumor signal transduction pathways demonstrate that aberrant activation of several molecules in various signaling pathways controlling cell cycle, proliferation, differentiation, cell survival, and apoptosis causes HCC progression [16]. 

#### 3.4.1. Wnt/β-Catenin Pathway

**Figure 4:**
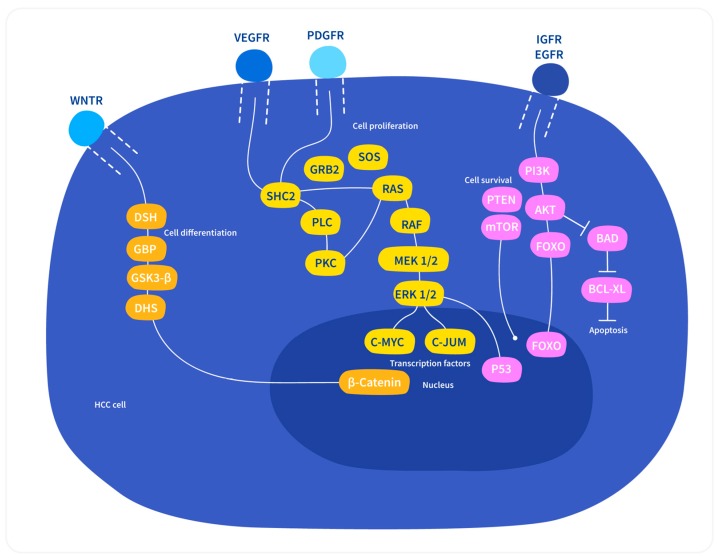
Potential cellular signaling pathways involved in the pathogenesis of hepatocellular carcinoma [28].

The Wnt/β-catenin signaling pathway is involved in maintaining cellular homeostasis via cell proliferation, differentiation, motility, and apoptosis [11]. The pathway comprises ligand Wnt protein, frizzled receptor, and regulator proteins, such as GSK-3β and β-catenin. The binding of activated Wnt with receptor allows for β-Catenin accumulation, followed by β-catenin transfer to the nucleus, where it activates LEF/TCF transcription factor (Figure 4) that controls the transcription of key cell cycle gene—Cyclin D [16]. Abnormal activation of Wnt/β-catenin pathway is associated with a number of cancers, including HCC [11,16]. Approximately 20%–40% of HCC shows mutations in this pathway. Frequently mutation occurs in the N-terminal of β-catenin that causes constitutive transcriptional activation of β-catenin/TCF complexes [12]. The upregulation of frizzled-7 gene and the dephosphorylation of β-catenin are also noticed in HCC [11]. Wnt/β-catenin pathway activation is correlated with HCV infection and AFB1 exposure. A lower frequency of β-catenin mutation occurs in HBV related HCC [12]. Additionally, mutations in Axin-1 and Axin-2, which negatively regulate the Wnt/β-catenin pathway, were observed in HCC [11]. All of these findings propose a critical role of Wnt/β-catenin signaling pathway in HCC development [11].

#### 3.4.2. Ras/Raf/MAPK Pathway

Ras/Raf/MAPK Pathway is the pivotal signal transduction pathway involved in HCC development. This pathway is normally responsible for cell proliferation, cell growth, differentiation, and survival. The upstream molecules of this pathway are different receptor tyrosine kinases, including Insulin-like growth factor receptor (IGFR), Vascular epidermal factor receptor (VEGFR), platelet-derived growth factor receptor (PDGFR), hepatocyte growth factor receptor (HCFR), and c-met receptor. The binding of the growth factors with these receptors cause phosphorylation and activation of the receptors and the signal is transduced to the downstream signaling pathway Ras/Raf/MAPK through Grb2/Shc/SOS molecules, and subsequently activates the transcription factor genes *c-myc*, *c-fos*, and *c-jun* that drive cell proliferation and cell growth [3,16] (Figure 4). The dysregulation of this pathway, due to aberrant upstream signals, inactivation of the Raf kinase inhibitor protein, and the presence of HBV and HCV proteins, results in anomalous cellular activity, leading to cancer. Ongoing research is attempting to discover effective drugs to block overexpressed Ras/Raf/MAPK signaling pathway in HCC [16]. 

#### 3.4.3. PI3/AKT/mTOR Pathway 

This pathway is involved in cell growth metabolism, survival regulation, and apoptosis. The activation of this pathway occurs in 30%–50% of HCC. The membrane lipid phosphatidylinositol 4,5-biphosphate (PIP2) is phosphorylated by PI3 kinase (PI3K), which binds to and activates serine threonine kinase Akt [28]. Tumor suppressor gene, PTEN, which targets the lipid products of PI3K for dephosphorylation, acts as a negative regulator of this pathway in normal cells. PTEN mutation decreases PIP3 level and overactivates the PI3/AKT/mTOR pathway (Figure 4), thus inhibiting apoptosis and inducing tumor development. The loss of PTEN and upregulation of p-AKT and p-mTOR are linked with tumor grade, vascular invasion, intrahepatic metastasis, and matrixmetalloprotease-9 upregulation [16,28].

#### 3.4.4. JAK/STAT Pathway

Janus Kinase (JAK) is a signal transducer and activator of a family of transcription factors STATs. The JAK/STAT pathway is activated by various cytokines and growth factors and it is involved in multiple cellular functions such as differentiation, proliferation, and apoptosis. Activated JAK triggers the transcription of *SOCS* genes, which belong to the negative feedback loop in the JAK/STAT pathway. The deregulation of the inhibitors of this pathway, particularly SOCS-1 and SS-1 (a JAK-binding protein), has been detected in HCC [11]. Studies showed that STAT3 is preferentially activated in human HCC and active STAT3 is linked with aggressive tumor phenotype [17].

#### 3.4.5. Ubiquitin-Proteasome (UP) Pathway

This is a highly conserved pathway in eukaryotes that degrades nearly 80% of cellular proteins. The ubiquitin molecules ligated sequentially ligated to form polyubiquitin chain on proteins that need to be degraded. 26S proteasome recognizes polyubiquitinated proteins and degrade them. Several tumor suppressor genes, some receptor tyrosine kinases, some oncogenes, and cell regulator molecules are controlled by the UP pathway [13]. This pathway is imperative for maintaining cellular homeostasis and its deregulation is a major contributory factor for myriad of diseases, including cancers [13]. Gankyrin, a subunit of 26S proteasome, has been routinely over expressed in human HCCs. The overexpression of gankyrin phosphorylates *pRb* and releases active E2F transcription factor driving more cell division. Moreover, a higher level of gankyrin increases the risk of polyubiquitination and the subsequent degradation of *p53* [32]. Mounting evidences about multiple roles of UP pathways in HCC pathogenesis point that it may be a hotspot on which novel therapies can be developed [13,38]. 

### 3.5. Angiogenesis and HCC

HCC is a hypervascularized tumor that greatly depends on angiogenesis. During advancement from early to moderately differentiated stage, angiogenesis occurs, which enables the malignant cells to invade vessels and metastasize [28]. Both angiogenesis and cell proliferation are involved in HCC initiation and progression [26]. The angiogenic switch of HCC is under the control of variety of angiogenic growth factors and inhibitors, including VEGF, angiopoietins, basic fibroblast growth factor (bFGF), TGF-α, and IGF-II. The most potent and crucial factor for promoting vessel growth and tumor progression is VEGF. The upregulation of VEGF and its receptors was detected in cirrhotic liver and they have been determined in HCC at both the tissue and serum levels. Higher VEGF level is linked with poor prognosis [8]. 

## 4. Molecular Targeted Therapies for HCC

The advancement in molecular cell biology over the last few decades improved our understanding of the detailed molecular mechanisms underlying tumor initiation and progression. This, in turn, provided opportunities to develop novel molecular-targeted agents, which restrain molecular abnormalities, as promising therapeutic interventions for cancer [39,40]. At present, many clinical trials are being conducted for finding agents that act on growth factor receptors and intracellular signaling pathways. 

### 4.1. Anti-Angiogenic Agents

Angiogenesis plays a central role in each step of hepatocarcinogenesis, and this is the reason why a current molecular-targeted therapeutic strategy for HCC mostly targets VEGF, among other angiogenic pathways, to develop potent anti-angiogenic agents [8,26]. 

#### 4.1.1. Sorafenib

This anti-angiogenic, multi-tyrosine kinase inhibitor was the first targeted, systemic therapy that was approved for the treatment of advanced HCC by Food & Drug Administration (FDA) in 2007 [39,41]. Sorafenib shows its anti-tumoral activity by blocking various receptor tyrosine kinases of the growth factors, including VEGF, PDGF, and c-Kit, thus inhibiting Raf/MEK/ERK mediated signal transduction [16]. Two global Phase III randomized controlled trials—SHARP and Asia-Pacific—detected the effectiveness of sorafenib in improving the overall survival of patients with unresectable and advanced HCC [42]. In both trials, the selected patients had advanced hepatocellular carcinoma, but had not received any systemic therapy before and had Child-Pugh A liver disease. The study participants received oral sorafenib (400 mg) or placebo twice daily. In SHARP trial median OS in sorafenib group was 10.7 months vs. 7.9 months in the placebo group, however median time-to-progression (TTP) did not significantly vary between the two groups (4.1 months vs. 4.9 months), whereas in the Asia-Pacific trial, both median OS and TTP are significantly higher in the sorafenib group than placebo group (6.5 months vs. 4.2 months and 2.8 months vs. 1.4 months) [43,44] (Table 1). The most common treatment-related adverse effects (AE) with sorafenib included diarrhea, hand-foot skin reaction, weight reduction, fatigue, and anorexia [43,44]. However, there was less probability of discontinuation of sorafenib due to AE [40], and the rate of discontinuation of the drug due to AE was analogous in both groups, as observed in SHARP trial [43]. With the success of sorafenib, additional clinical trials were conducted to assess other molecular targeted agents with the goal of improved safety/efficacy when compared to sorafenib [41,42]. Two superiority trials comparing sorafenib with sunitinib (SUN 1170 trial) and linifanib versus sorafenib (LiGHT), both drugs that primarily target VEGFR and PDGFR showed that sunitinib was not superior to sorafenib in terms of the primary endpoint of overall survival (OS) (8.1 months for sunitinib vs. 10.0 months for sorafenib, *p* = 0.0019); linifinib also failed to meet its primary endpoint of superiority in overall survival (9.1 months for linifanib vs. 9.8 months for sorafenib), and it was found to be linked with more grade 3 or 4 adverse events than sorafenib [41,42] (Table 1). Another superiority trial- BRISK-FL (phase III, randomized study) with brivanib (which targets VEGFR, PDGFR, and FGFR) vs. sorafenib failed to prolong OS (9.5 months for brivanib vs. 9.9 months for sorafenib, *p* > 0.05) [41]. However, the toxicity profile of brivanib was better than sorafenib [41] and it was reported to be effective for sorafenib-resistant HCC [39] in a placebo-controlled study- BRISK-PS. Brivanib treatment did yield an increased median TTP, but it did not significantly increase the OS (9.4 months vs. 8.2 months, *p* = 0.33) [45]. The antitumor activity of both agents was alike and the safety profile of brivanib was acceptable [45]. However, the study failed to meet the primary endpoint of OS noninferiority for brivanib versus sorafenib, and because of that brivinab is not used to treat HCC in USA. Hence, sorafenib remained the only FDA-approved TKI for the treatment of HCC [16].

#### 4.1.2. Lenvatinib 

For over a decade, sorafenib remained as the only FDA-approved first-line systemic treatment for advanced, unresectable HCC. Lenvatinib, a multikinase inhibitor of VEGF receptors 1–3, FGF receptors 1–4, PDGF receptor α, RET, and KIT, was assessed in a phase II study, which found that lenvatinib showed clinical activity and satisfactory safety profile in unresectable HCC (uHCC). This led to a phase III randomized, open-label, non-inferiority clinical trial of lenvatinib vs. sorafenib in first-line treatment for uHCC; lenvatinib treatment resulted in statistically significant and clinically meaningful improvements in TTP and progression free survival (PFS), and met its primary endpoint of noninferiority to sorafenib in terms of OS (9.1 months vs. 9.8 months) [46]. The results of this phase III trial were initially presented at ASCO 2017, which opened a new option for first-line molecular targeted therapy. The REFLECT trial (NCT01761266), a multicenter, international, randomized, open-label, non-inferiority phase III trial, was conducted on patients with uHCC (Table 1). This study compared lenvatinib versus sorafenib as a first-line treatment. The inclusion criteria of this study were patients with uHCC (confirmed histologically or cytologically) and Child-Pugh A liver disease with adequate liver function and controlled blood pressure. The patients received 12 mg/day or 8 mg/day lenvatinib, depending on body weight or sorafenib 400 mg twice daily in 28-days cycle. The primary endpoint was OS and secondary outcomes were TTP and progression free survival (PFS). Median OS for lenvatinib of 13.6 months was non-inferior to sorafenib of 12.3 months. TTP was 7.4 months for lenvatinib and 3.7 months with sorafenib [47]. An improvement in PFS was observed with lenvatinib rather than sorafenib. Some of the treatment-emergent AEs were more frequent with lenvatinib than sorafenib, including hypothyroidism, hypertension, proteinuria, dysphonia, and decreased body weight. In contrast, the common AEs with sorafenib were hand-foot skin reaction, diarrhea, palmar-plantar erythrodysaesthesia, alopecia, and reduced appetite [42,47]. This clinical trial indicated that lenvatinib had a significantly better antitumor effect than sorafenib [40]. Lenvatinib became the second TKI to attain FDA approval for the first-line treatment of patients with uHCC based on the findings of REFLECT trial, in August 2018 [48].

#### 4.1.3. Regorafenib

Regorafenib is a multikinase inhibitor that has close structural similarity with sorefenib. Regorafenib inhibits VEGFR2,3, PDGFR, FGFR-1, Kit, Ret, and B-Raf [49]. The use of regorafenib vs. placebo as a second-line therapy following sorafenib failure was studied in a randomized, double-blind, phase III clinical trial (RESORCE trial, NCT01774344). The patients that were selected for this study had HCC confirmed pathologically or via non-invasive evaluation. They had Child-Pugh A liver function and must have tolerated sorafenib ≥400 mg/day for a minimum 20 of the 28 days prior to discontinuation and they had received their last sorafenib dose within 10 weeks of randomization. The patients receiving any prior systemic treatment for HCC or stopped taking sorafenib for toxicity were excluded from the study. The study participants received oral 160 mg regorafenib/day for the first three weeks of each 28-day cycle initially. In all regorafenib recipients, AEs were observed and the most frequent grade 3 or 4 AEs were hypertension, hand-foot skin reaction, fatigue, and diarrhea. The most common treatment-related AE leading to the discontinuation of regorafenib were elevated aspartate aminotransferase (AST) and alanine aminotransferase (ALT) concentrations and hand-foot skin reaction [50]. To control toxicity, the dose was reduced to 80 mg/day and the requirement of any further dose reduction led to treatment discontinuation [50]. The study determined that regorafenib had improved OS significantly; median OS for regorafenib group experiencing radiologic progression during sorafenib therapy was 10.6 months, in contrast to 7.8 months for placebo (*p* < 0.0001) [50]. The safety profile of regorafenib was quite comparable. Based on the RESORCE trial data, the FDA approved regorafenib as second-line treatments for HCC in patients progressing on sorafenib who are not eligible for alternative treatment [49]. Future trials are exploring combinations of regorafenib with other systemic agents as third-line treatment for patients who are unable to sequentially tolerate sorafenib and regorafenib [50].

#### 4.1.4. Cabozantinib

Cabozantinib is a multiple tyrosine kinase inhibitor that inhibits VEGFR 1-3, MET, and AXL. A double-blind, phase III clinical trial (CELESTIAL trial, NCT01908426) was conducted on patients with uHCC who had progressed on sorafenib and did not respond to any curative treatment. The selected patients were of age 18 years or more and have Child-Pugh Class A liver function without any uncontrolled clinically significant illness. The advanced HCC patients with Child-Pugh Class B liver function were excluded from the study. The median average dose of cabozantinib per day was 35.8 mg. The study demonstrated a significantly longer median OS with cabozantinib when compared to the placebo (10.2 months vs. 8.0 months, *p* = 0.005), with a median PFS of 5.2 months and 1.9 months of cabozantinib vs. placebo, respectively [51]. Most common grade 3 or 4 AEs included palmar-plantar erythrodysesthesia, fatigue, reduced appetite, nausea, and diarrhea. Grade 5 AEs reported in few patients in cabozantinib group were hepatic failure, portal-vein thrombosis, hepatorenal syndrome, and pulmonary embolism [51]. Based on the data of CELESTIAL trial, in January 2019, the FDA approved cabozantinib as second-line treatment option for advanced HCC patients who had previously been treated with sorafenib [52]. 

### 4.2. EGFR Inhibitors 

A superiority trial of sorafenib vs. erlotinib in a phase III, randomized, controlled, double-blind trial (SEARCH trial) failed to meet its primary endpoint, as it neither prolongs TTP (3.2 months vs. 4.0 months, *p* > 0.05) nor OS (9.5 months vs. 8.5 months; *p* > 0.05) [41]. A phase II study with bevacizumab (anti-VEGF monoclonal antibody) plus erlotinib in the treatment of advanced HCC patients resulted in little antitumor activity as compared to the control arms, who were receiving either sorafenib or bevacizumab [53]. 

### 4.3. mTOR Inhibitors

Aside from targeting angiogenic, especially VEGFR, inhibitors, there are several clinical trials that aimed to identify additional unique molecular therapeutic agents targeting other inhibitors. Everolimus (RAD001), which inhibits mTOR (another critical target implicated in hepatocarcinogenesis) has been extensively studied for the treatment of HCC [50]. A randomized, double-blind, phase-III clinical trial—EVOLVE-1—was conducted to study the effect of everolimus on patients previously treated with or intolerant to sorefenib. The study detected no significant difference in OS between the evorolimus treated group vs. placebo (OS 7.6 months vs. 7.3 months, respectively) and the median TTP with everolimus and placebo was 3.0 months and 2.6 months, respectively. Based on of this, everolimus is not an approved current treatment option for patients with advanced HCC during or after receiving sorafenib [54]. However, there are a few ongoing phase I and II trials studying the effects of everolimus on patients with HCC, either as a single agent or combination with sorafenib or a cytotoxic agent, such as doxorubicin.

### 4.4. c-MET Inhibitors

c-Met signaling plays role in hepatocarcinogenesis. Several c-MET inhibitors have been studied in several trials. Foretinib (GSK 136089), the first multi-tyrosine kinase inhibitor targeting c-MET, was investigated clinically and found with a TTP of 4.2 months and median OS of 15.7 months in sorafenib-naïve HCC patients. Trivantinib, another competitive inhibitor of c-MET, was tested in a phase-III, randomized, placebo-controlled trial, failed to improve OS compared to placebo in patients with MET-high advanced HCC already treated with sorefenib [55]. Phase II and phase III clinical trial data indicated c-MET inhibitors are usually well tolerated, with the exception of increased occurrence of grade 3 or 4 neutropenia [41]. Further randomized studies are required to establish whether inhibition of c-MET receptor could be a potential therapeutic agent for selected patients with advanced HCC [55].

### 4.5. MEK Inhibitors

Selumetinib (AZD6244), which is a MEK inhibitor, was found to result in a short TTP of 1.8 months in a phase II trial with treatment-naïve HCC patients [41]. A recent phase Ib study using a combination of selumetinib and sorafenib was conducted on patients of Asian ethnicity with advanced HCC. The median OS was 14.4 months with acceptable adverse events and encouraging anti-tumor activity in this population [56].

### 4.6. Other Molecular Targeted Agents

Two superiority trials compared sorafenib with radioembolization, SARAH and SIRveniB, in locally advanced HCC, and they were also reported at EASL2017 and ASCO 2017; however, these trials failed to meet their primary endpoints, with one criticism being the difficulty in performing clinical-trials of first-line HCC while using OS as the endpoint. A phase I study with TAC-101, an oral synthetic retinoid, with Japanese HCC patients showed positive anti-tumor activity with a satisfactory toxicity profile [57]. There is an ongoing phase III (NCT00756782) study that combines TAC-101 and transcathetar arterial chemoembolization (TACE) vs. TACE alone in Japanese HCC patients [58]. 

### 4.7. Immunotherapy for HCC Treatment

The escape from immunological surveillance is a hallmark for tumor progression. The identification of immune checkpoint molecules has provided the rationale for development of immunotherapy in HCC. These drugs suppress an immune checkpoint that may be used by tumor cells to protect themselves from and evade the immune system, and thus this treatment is used to treat a variety of carcinomas with variable success. Immunotherapy approaches by blocking immune checkpoint revealed initial encouraging results in advanced HCC [59]. Currently, positive trials with the use of monoclonal antibodies pembrolizumab and nivolumab targeting programmed death-1 (PD-1)/programed death ligand-1 (PDL-1) have been completed [7].

#### 4.7.1. Pembrolizumab

Pembrolizumab, an anti-PD-1 monoclonal antibody, exhibits effective antitumor activity and a manageable safety profile in multiple cancers. In a phase II open-label clinical trial (Keynote-224 trial, NCT02702414) that was conducted to evaluate the safety and efficacy of pembrolizumab in advanced HCC patients previously treated with sorafenib who experienced either disease progression or intolerance to treatment, pembrolizumab showed durable responses along with favorable PFS (4.8 months), median OS (12.9 months), and TTP (4.9 months) in HCC patients. Its safety profile is comparable to that which has been previously established for pembrolizumab monotherapy [56]. The most frequent AEs included enhanced ALT and AST levels, hypothyroidism, and skin rashes. Immune-mediated events were observed in some participants, such as hypothyroidism, adrenal insufficiency, and rarely Type I diabetes mellitus [60]. The results of this study led to a phase III double-blind, randomized, controlled trial (Keynote-240 trial, NCT0270240) that compared pembrolizumab with best supportive care versus placebo with best supportive care as second-line therapy for patients with HCC who previously received systemic therapy [61]. However, the study failed to meet primary endpoints, as the patients that were treated with pembrolizumab did not show statistically significant higher OS and PFS when compared to the placebo group. The safety profile was similar to previous studies [61]. Additional ongoing trials are currently under evaluation for other immunotherapy agents. Immunotherapy can be considered for HCC patients who are unable to withstand multikinase inhibitors or that have deteriorated liver function. 

#### 4.7.2. Nivolumab

A checkpoint inhibitor has been conditionally approved by FDA in September 2017 as a second-line treatment of individuals with HCC who had been previously treated with TKIs. The approval was based on the data of the multi-cohort, open, non-comparative, phase 1/2 trial—Checkmate 040 (NCT01658878). This trial included advanced uHCC patients who showed progression upon being treated with one first line systemic therapy or intolerant to sorafenib and had Child-Pugh A liver condition. The median OS was 15.1 months and median TTP was 4.1 months. The treatment yielded a manageable safety profile and promising efficacy, which indicated that nivolumab had significant benefits for pretreated patients [62]. A phase III clinical trial CheckMate-459 (NCTC2576509) is now underway to explore the efficacy of nivolumab as a first-line therapy for advanced uHCC. The primary outcome of this trial is OS and PFS is the secondary outcome. The preliminary data of this trial after primary completion date (October 2018) revealed OS, PFS, and overall response rate (ORR) to be approximately 33 months. However, the full reporting of this trial is still awaiting [63]. 

#### 4.7.3. Bevacizumab

A recombinant humanized monoclonal antibody that was directed against VEGF had been studied as a single agent or combination therapy in a phase II study with uHCC patients. The six-month PFS was found to be 65% and OS at 1, 2, and 3 years was 53%, 28%, and 23%, respectively. A combination of bevacizumab with gemcitabine and oxaliplatin resulted in 20% overall response rate and the median OS was 9.6 months [53].

#### 4.7.4. Ramucirumab

A recombinant human IgG1 monoclonal antibody inhibiting the ligand activation of VEGFR2 and having antitumor activity had been investigated in a double blind, placebo-controlled, randomized, global, phase III trial—REACH 2 (NCT02435433)—as the therapeutic agent for advanced HCC patients that were previously treated with first-line sorafenib. The study participants had confirmed HCC with Child-Pugh class A liver disease and serum α-fetoprotein (AFP) concentration of 400 ng/mL or more and showed intolerance or disease progression following sorafenib treatment, and thus discontinued the medicine. The patients received 8 mg/kg ramucirumab and best supportive care (BSC) or placebo with BSC every two weeks. The primary outcome OS was markedly higher in ramucirumab group (8.5 months) when compared to placebo group (7.3 months). The secondary outcome PFS was prolonged in the ramucirumab group (2.8 months) than the placebo group (1.6 months). The most frequent AEs with ramucirumab included fatigue, hypertension, peripheral edema, abdominal pain, loss of appetite, proteinuria, and nausea [64]. Based on the findings of REACH-2, on 10 May 2019 FDA approved ramucirumab as a single agent to treat HCC patients with AFP concentration ≥400 ng/mL and had prior treatment with sorafenib. The recommended ramucirumab dose is 8mg/kg in every two weeks [65]. 

### 4.8. Immunotherapy in Adjuvant Setting

An increased rate of recurrence of HCC, even after curative resection or ablation, poses a major threat in improving patient prognosis, which suggests the importance of adjuvant therapies for HCC patients. Nonetheless, several adjuvant therapies showed failure in terms of OS and recurrence-free survival (RFS). Several trials that were conducted with Interferons (IFNs) and other immunotherapies in adjuvant setting produced heterogeneous and ambiguous results [66]. Furthermore, adjuvant therapy following curative treatment has not been encouraged by the latest international practice guidelines [67]. A recent multicenter, open-labeled, randomized controlled trial by Lee et al. revealed adjuvant adoptive immunotherapy while using autologous cytokine (CIK) cells (polyclonal T-lymphocytes that grow rapidly and possess strong antitumor effect) extended significantly both OS and RFS of the patients who underwent possible curative treatment for HCC. The study results are quite encouraging and suggested that the patients receiving curative therapy for early stage HCC are ideal for immunotherapy in adjuvant setting [67]. The efficacy of nivolumab as an adjuvant therapy following surgical resection or ablation therapy of HCC is presently being evaluated in a phase III trial—CheckMate 9DX (NCT03383458). The patients with the highest risk of recurrence are included in this study and their treatment will be continued until recurrence, so as to compare the RFS period as the primary endpoint. Currently (as of September 2018), this is the sole phase III trial using nivolumab as an adjuvant therapy in HCC patients [68]. The results of this trial are pending.

## 5. Challenges in Treatment of HCC Patients 

HCC is an aggressive malignancy with rising incidence globally. The management of patients with HCC is complex, particularly as it is imperative to consider both tumor stage and underlying liver disease simultaneously. The majority of patients with cirrhosis are diagnosed at an advanced stage [69]. Advanced HCC patients show diverse clinical conditions and radiological features [69] and the treatment decisions depend on clinical stage, liver function, and patient factors. Cytotoxic chemotherapy agents and immunotherapy provided marginal efficacy in HCC [26]. In the past 20 years, few clinical trials were conducted to examine the efficacy of hormonal treatment in HCC patients. One of the largest trials used anti-estrogen tamoxifen as systemic treatment, but failed to detect any survival advantage of tamoxifen. Negative results were also obtained with anti-androgen therapy. No concrete evidence was available for contemplating HCC as a hormone-responsive tumor and, hence, hormonal therapy is not considered a part of HCC management at present [70]. Recently, molecular targeted therapies are showing promising results for the treatment of advanced uHCC [16]. The approval of sorafenib as a first-line systemic therapy for HCC was a major breakthrough in HCC treatment. Following that, a plethora of trials studying a range of drugs for second-line treatment for HCC were conducted in the last decade and a majority of these studies had negative results. Nevertheless, a considerable improvement in HCC treatment options with successful results has been observed currently, as shown in Table 1. 

## 6. Conclusions with Future Directions 

HCC is a complicated disease with an overall poor prognosis in advanced stage. Numerous signaling pathways contribute to the disease pathogenesis. Molecular targeted therapies, which inhibit specific growth factor receptors and their downstream signaling cascades, seem to be a favorable approach for management of HCC. Sorafenib is a revolutionary molecular-targeted drug showing effective results in many patients with advanced HCC. However, the majority of HCC patients who tolerated sorefenib showed disease progression. Numerous clinical trials have been undertaken to identify the effective drugs for this patient population. Currently, the FDA has approved four additional drugs—regorafenib, lenvatinib, cabozantinib, and as recent as ramucirumab—for the treatment of advanced HCC. Recent results of additional trials (in past year) have provided additional potential therapeutic options in this difficult disease (as referenced above). Furthermore, investigations are going on to detect molecular-targeted agents directed against other new pathways, particularly the apoptosis pathway.

To significantly improve HCC prognosis, further research is required to better understand the molecular mechanism of HCC and to identify other novel molecular targets for the effective intervention of advanced HCC. Besides monotherapy, combination therapy, either with multiple targeted agents or targeted therapy, along with traditional chemotherapy, might be a more effective modality to treat HCC. Many clinical trials of novel agents, as well as combination therapy for HCC, are currently underway, with the potential for bringing drastic changes in the treatment of advanced HCC in the coming year.

## Figures and Tables

**Table 1:** Clinical trials of molecular targeted therapy in hepatocellular carcinoma registered at clinicaltrials.gov.

Treatment	Target	Trial Phase, Name, clinicaltrial.gov Number	OS	TTP	Results	Ref.
Sorafenib	Multi-kinase inhibitor	Phase III vs. placebo (SHARP; NCT00105443)	10.7 months vs. 7.9 months (*p* < 0.001)	4.1 months vs. 4.9 months (*p* = 0.77)	Positive	[43,44]
		Phase III vs. placebo (Asia-Pacific; NCT00492752)	6.5 months vs. 4.2 months (*p* = 0.014)	2.8 months vs. 4.9 months (*p* = 0.0005)		
Sunitinib	VEGFR, PDGFR inhibitor	Phase III vs. sorafenib (SUN 1170; NCT00699374)	8.1 months vs. 10.2 months (two-sided *p* = 0.019)	4.1 months vs. 3.8 months (two-sided *p* = 0.3082)	Negative	[41,42]
Linifanib	VEGFR, PDGFR inhibitor	Phase III vs. sorafenib (LIGHT; NCT01009593)	9.1 months vs. 9.8 months (*p* = NS)	5.4 months vs. 4.0 months (*p* = 0.001)	Negative	[41,42,46]
Brivanib	VEGFR, PDGFR, FGFR inhibitor	Phase III vs. sorafenib (BRISK-FL; NCT00858871)	9.5 months vs. 9.9 months (*p* = 0.3116)	4.2 months vs. 4.1 months (*p* = 0.853)	Negative	[41,42]
Lenvatinib	Multi-kinase inhibitor	Phase III vs. sorafenib (REFLECT; NCT01761266)	13.6 months vs. 12.3 months	8.9 months vs. 3.7 months (*p* < 0.0001)	Positive	[47]
Regorafenib	Multi-kinase inhibitor	Phase III vs. placebo (RESORCE; NCT01774344)	10.6 months vs. 7.8 months (*p* < 0.0001)	-	Positive	[50]
Cabozantinib	Multi-kinase inhibitor	Phase III vs. placebo (CELESTIAL; NCT01908426)	10.2 months vs. 8.0 months (*p* = 0.005)	-	Positive	[51]
Erlotinib	EGFR inhibitor	Phase III vs. sorafenib (SEARCH; NCT00901901	9.5 months vs. 8.5 months (*p* > 0.05)	3.2 months vs. 4.0 months (*p* > 0.05)	Negative	[41]
Everolimus	mTOR inhibitor	Phase III vs. placebo (EVOLVE-1; NCT01035229)	7.6 months vs. 7.3 months	3.0 months vs. 2.6 months	Negative	[54]
Trivantinib	c-Met inihibitor	Phase III vs. placebo (NCT01755767)	8.4 months vs. 9.1 months (*p* = 0.81)	-	Negative	[55]
Selumetinib	MEK inhibitor	Phase II vs. placebo (NCT00604721)	-	-	-	[41]
		Phase Ib vs. sorafenib	14.4 months with selumetinib	-		
Pembrolizumab	Anti-PD-1 monoclonal antibody	Phase II (KEYNOTE-224; NCT02702414)	12.9 months	4.9 months	Negative	[61]
		Phase III vs. placebo (KEYNOTE-240; NCT02702401)	-	-		
Bevacizumab	Anti-VEGF antibody	Phase II vs. placebo	53% (1 year) 28% (2 years) 23% (3 years)	-	Negative	[53]
Bevacizumab + Gemcitabine + Oxaliplatin		Phase II	9.6 months	-	Negative	[53]
Nivolumab	Anti-PD-1 monoclonal antibody	Phase I/II (CheckMate-040; NCT01658878)	15.0 months	3.4 months	Positive	[62]
Ramucirumab	Human IgG1 monoclonal antibody	Phase III vs. placebo (REACH-2; NCT02435433)	8.5 months vs. 7.3 months (*p* = 0.0199)	Median time to radiologic progression 3.0 months vs. 1.6 months (*p* < 0.0001)	Positive	[64]
